# Dataset on the road traffic noise measurements in the municipality of Thessaloniki, Greece

**DOI:** 10.1016/j.dib.2020.105214

**Published:** 2020-01-31

**Authors:** Paraskevi Begou, Pavlos Kassomenos, Apostolos Kelessis

**Affiliations:** a Laboratory of Meteorology, Department of Physics, University of Ioannina, GR-45110, Ioannina, Greece; b Environmental Department, Municipality of Thessaloniki, Paparigopoulou 7, Thessaloniki 54630, Greece

**Keywords:** Noise pollution, Road traffic noise, L_eq_, L_den_, L_night_

## Abstract

The continuous monitoring of environmental noise levels is deemed necessary, such in the strategic noise mapping or in the cohort studies. The environmental noise levels can be measured and analysed with the aid of various methods. In this article presents the method recommended by the European Union (EU). The data contain the road traffic noise level measurements in the Greater Thessaloniki area, Greece. The L_eq_ noise measurements carried out at two different locations, the Urban Highway-Hot Spot area and the Residential area, for a 3 year measurement period. Also, the analysis was based on the environmental noise indicators L_day_, L_den_, L_evening_, L_night_.

**Table:** Specification Table

Subject	Acoustics
Specific subject area	Noise measurements, Environmental Noise, Road Traffic Noise
Type of data	Tables, Figures, Graphs
How data was acquired	The road traffic noise measurements were carried out at two selected locations for a 3 year period, using sound measuring instruments
Data format	Raw, filtered and analyzed
Parameters for data collection	The sound level meter records continuous steady noise levels during a 24-h period
Description of data collection	The measurements were performed with a noise level analyzer, type Solo Master (01dB-Stell-ΜVI Technologies Group, France) and with a outdoor microphone, type MCE 212 (class 1). The data were recorded with a computer, which was equipped with the software.
Data source location	Environmental Department of Municipality of Thessaloniki, Greece Latitude and longitude for collected data: Egnatia street (Highway-Hot Spot area): 40°38′16″N, 22°56′29″E 25th Martiou street (Residential area): 40°36′05″N, 22°57′36″E
Data accessibility	The data are available in the article and as a supplementary file that can be found in the online version
Related research article	Begou P., Kassomenos P. And Kelessis A., Effects of road traffic noise on prevalence of cardiovascular diseases: The case of Thessaloniki, Greece, Science of the total Environment, 2020 https://doi.org/10.1016/j.scitotenv.2019.134477

**Table:** 

**Value of the data** • The data base provides an extensive picture of the road traffic noise in the urban area of Greater Thessaloniki Area. • The dataset gives the opportunity to researchers for comparing and validating the noise assessment methods with the actual noise measurements. • The data can be used to develop the strategic noise mapping and action plans. • The noise metrics are essential for the policy makers and risk managers to judge the necessity of taking noise prevention methods or mitigation measures and to evaluate the effectiveness of these measures. • The data can help to establish the environmental noise limit values for the health protection.

## 1 Data description

The environmental noise pollution is one of the major public issues affecting the inhabitants in the Greater Thessaloniki Area, in Greece.

The Environmental Department of Municipality of Thessaloniki performed systematic measurements of the urban road traffic noise levels. These measurements, mainly, were performed in commercial or residential areas in busy roads with high traffic load. The dataset from these recordings is provided as a supplementary material in the online version of the article.

**Fig. 1:**
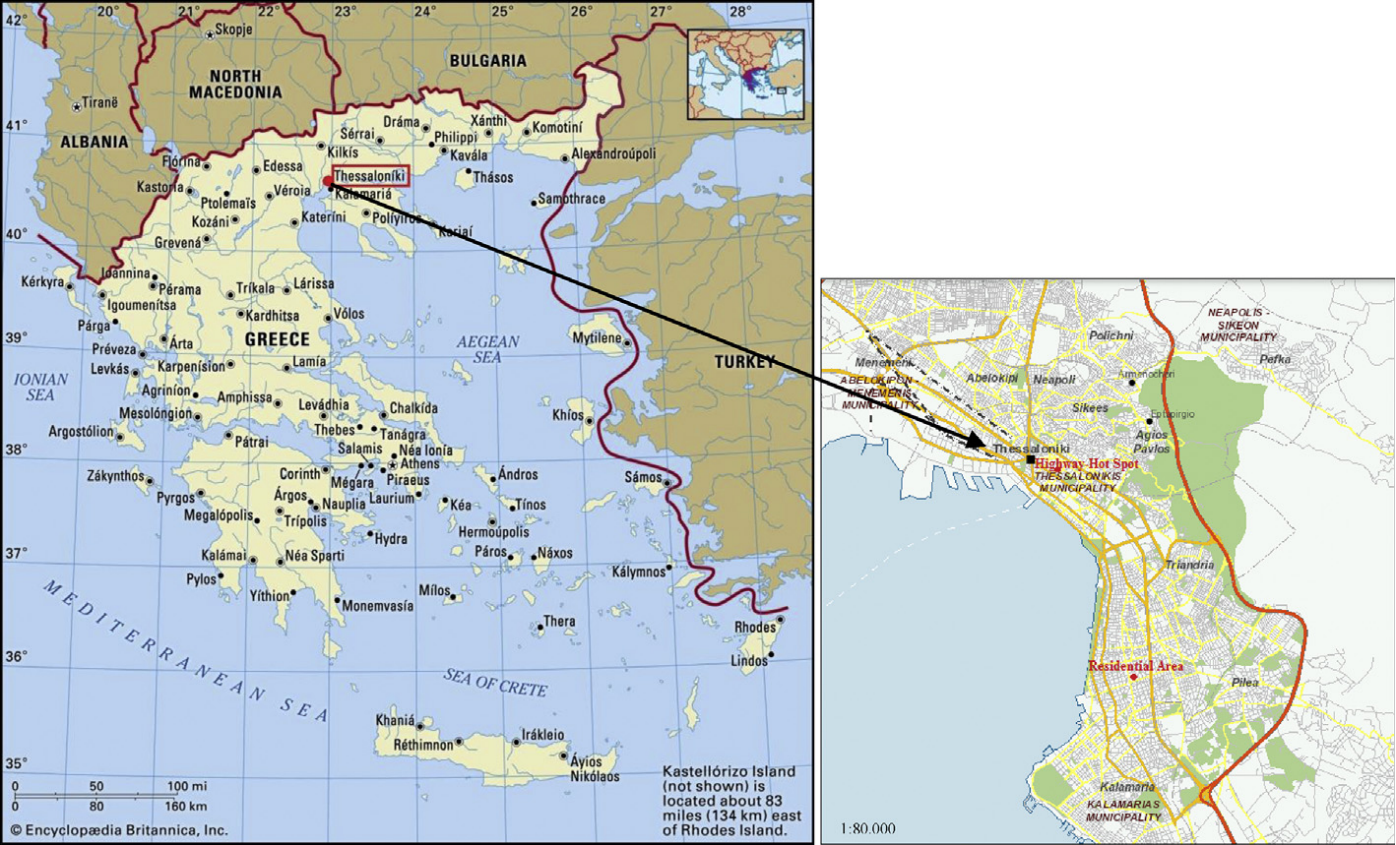
Location of the measurement areas in the Greater Thessaloniki Area.

The data presented in this article contain the road traffic measurements at two different locations in the urban area of Thessaloniki. The choice of these two areas arose from the fact that they experience high volumes of road traffic. The locations of the measurement points are presented in Fig. 1. The measurements were performed in two investigated areas, at Egnatia street and 25th Martiou street. Therefore, Egnatia and 25th Martiou streets named Highway-Hot Spot and Residential area, respectively. The Highway-Hot Spot area is the most influencing road in the city with the highest traffic load and the maximum vehicle fleet. Similarly, the Residential area represents one of the most noisy streets of the city with wide range of urban conditions.

Table 1, Table 2 summarizes the noise metrics mentioned above. The mean hourly values of the road traffic noise levels, which presented in Tabled 1, were collected in the Urban Highway-Hot Spot area from the annual measurements. Similarly, Table 2 presents the same values for the Residential area.

**Table 1:** The L_eq_ (dB(A)) road traffic noise measurements with standard deviation (SD) values for the Urban Highway-Hot spot area.

Measurement Period			Measurement Period			Measurement Period		
1/1/2004-31/12/2004			1/1/2005-31/12/2005			1/1/2006-31/12/2006		
Hour (EET)*	L_eq_ (dB(A))	SD (dB(A))	Hour (EET)*	L_eq_ (dB(A))	SD (dB(A))	Hour (EET)*	L_eq_ (dB(A))	SD (dB(A))
0:00	70.48	1.99	0:00	70.31	1.38	0:00	69.62	1.40
1:00	69.70	1.75	1:00	69.48	1.41	1:00	68.76	1.59
2:00	68.69	1.78	2:00	68.59	1.63	2:00	67.78	1.82
3:00	68.14	1.48	3:00	68.11	1.91	3:00	66.86	1.84
4:00	69.28	1.10	4:00	68.89	1.44	4:00	67.97	1.46
5:00	71.38	1.07	5:00	70.81	1.20	5:00	70.08	1.38
6:00	72.16	1.01	6:00	71.71	1.00	6:00	70.96	1.20
7:00	72.01	0.90	7:00	71.78	0.96	7:00	71.20	1.61
8:00	71.88	0.90	8:00	71.69	0.90	8:00	71.09	1.25
9:00	71.88	0.90	9:00	71.80	1.23	9:00	71.16	1.61
10:00	71.93	1.01	10:00	71.81	1.29	10:00	71.17	1.43
11:00	72.10	0.91	11:00	71.82	0.95	11:00	71.27	1.25
12:00	72.16	0.92	12:00	71.89	0.90	12:00	71.38	1.28
13:00	72.11	0.86	13:00	71.98	0.94	13:00	71.52	1.47
14:00	72.14	0.81	14:00	72.01	0.93	14:00	71.41	1.17
15:00	72.20	0.84	15:00	71.95	0.87	15:00	71.38	1.06
16:00	72.27	0.85	16:00	72.04	0.97	16:00	71.44	1.15
17:00	72.19	0.90	17:00	71.97	0.94	17:00	71.36	1.15
18:00	72.13	1.13	18:00	71.84	0.86	18:00	71.19	1.17
19:00	72.11	1.09	19:00	71.91	0.96	19:00	71.19	1.12
20:00	72.09	0.90	20:00	71.88	0.94	20:00	71.19	1.14
21:00	72.13	1.21	21:00	71.82	0.92	21:00	71.20	1.29
22:00	71.86	1.16	22:00	71.52	0.95	22:00	70.89	1.26
23:00	71.33	1.82	23:00	71.05	1.29	23:00	70.35	1.32

*(EET): Eastern European Time, UTC+02:00 time zone and UTC+03:00 during summer.

**Table 2:** The L_eq_ (dB(A)) road traffic noise measurements with standard deviation (SD) values for the Residential area.

Measurement Period			Measurement Period			Measurement Period		
1/1/2006-31/12/2006			1/1/2007-31/12/2007			1/1/2008-31/12/2008		
Hour (EET)*	L_eq_ (dB(A))	SD (dB(A))	Hour (EET)*	L_eq_ (dB(A))	SD (dB(A))	Hour (EET)*	L_eq_ (dB(A))	SD (dB(A))
0:00	65.60	1.56	0:00	64.49	2.55	0:00	64.74	2.45
1:00	65.34	3.19	1:00	63.64	2.77	1:00	63.77	2.19
2:00	63.74	2.56	2:00	62.52	2.31	2:00	62.61	2.38
3:00	62.64	1.86	3:00	62.44	1.84	3:00	61.70	2.56
4:00	62.71	1.48	4:00	63.52	2.49	4:00	61.76	2.41
5:00	64.32	2.03	5:00	65.02	2.24	5:00	63.81	2.15
6:00	65.88	1.35	6:00	66.11	2.04	6:00	65.15	2.02
7:00	66.97	1.95	7:00	66.78	2.26	7:00	66.08	2.08
8:00	66.88	1.34	8:00	66.76	1.80	8:00	66.45	2.29
9:00	67.71	2.24	9:00	67.07	1.93	9:00	66.80	2.48
10:00	68.15	2.59	10:00	67.34	2.21	10:00	66.92	2.31
11:00	67.76	2.12	11:00	67.99	2.90	11:00	67.38	2.86
12:00	68.14	3.37	12:00	67.31	1.79	12:00	67.07	2.37
13:00	68.33	2.74	13:00	67.57	2.58	13:00	67.25	2.46
14:00	67.74	2.30	14:00	67.35	2.53	14:00	66.94	2.39
15:00	67.14	1.11	15:00	67.26	2.42	15:00	66.85	2.52
16:00	67.40	1.88	16:00	67.00	2.27	16:00	66.76	2.29
17:00	67.25	1.42	17:00	66.74	1.98	17:00	67.21	3.22
18:00	67.08	2.21	18:00	66.95	2.46	18:00	66.38	2.36
19:00	66.89	2.11	19:00	66.93	2.53	19:00	66.50	2.41
20:00	67.13	2.48	20:00	66.88	2.48	20:00	66.50	2.31
21:00	67.15	2.43	21:00	66.37	1.79	21:00	66.40	2.39
22:00	66.75	1.93	22:00	65.87	2.13	22:00	66.57	2.94
23:00	66.30	1.52	23:00	65.26	2.19	23:00	65.71	2.47

*(EET): Eastern European Time, UTC+02:00 time zone and UTC+03:00 during summer.

The L_min_ and L_max_ values presented in Table 3. These values determined the hourly recordings of L_eq,T_, which calculated during the period of noise measurements.

**Table 3:** Minimum (L_min_) and maximum (L_max_) noise levels at the measurement locations.

Urban Highway-Hot Spot	L_min_ (dB(A))	Date	Hour (EET)*	L_max_ (dB(A))	Date	Hour (EET)*
2004	64.90	27 July 2004	2:00	89.20	05 July 2004	00:00
2005	63.90	15 August 2005	2:00	86.20	11 May 2005	10:00
2006	63.20	15 August 2006	3:00	86.00	07 September 2006	7:00
Residential Area	L_min_ (dB(A))	Date	Hour (EET)*	L_max_ (dB(A))	Date	Hour (EET)*
2006	58.90	27 September 2006	3:00	85.00	04 November 2006	12:00
2007	58.00	21 June 2007	3:00	82.90	28 November 2007	14:00
2008	56.90	30 June 2008	3:00	81.50	17 May 2008	11:00

*(EET): Eastern European Time, UTC+02:00 time zone and UTC+03:00 during summer.

**Fig. 2:**
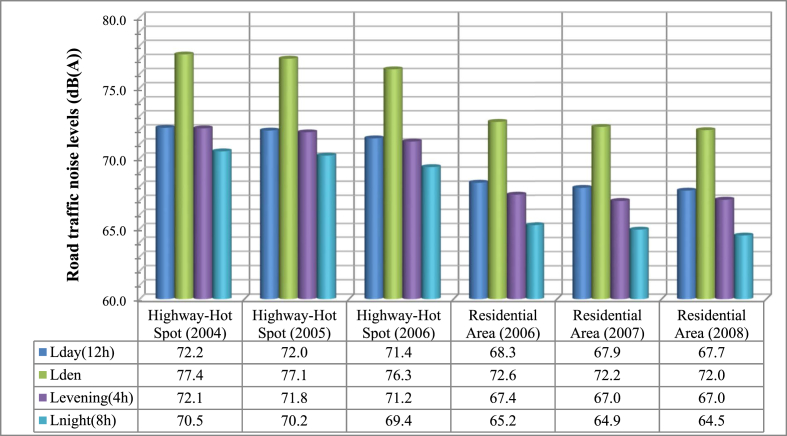
Road traffic noise levels estimated with L_day(12)_, L_den_, L_evening(4h)_,L_night(8h)_ indicators for the measurements locations during a 3-year period.

From Fig. 2, we can see the road traffic noise indicators, L_day_, L_den_, L_evening_ and L_night_ for the two measurement locations for a 3 year time period.

The details from the aforementioned road traffic noise measurements are discussed in Begou et al. (2020). In the same work the relation of road traffic with the Greek financial crisis is investigated. Also, they estimated the prevalence of cardiovascular diseases due to exposure to road traffic noise is estimated.

## 2 Experimental design, equipment and analyses

### 2.1 Noise exposure assessment

The road traffic noise measurements are carried out using the recommended method, in accordance to the requirements of the EU Environmental Directive 2002/49/EC.

Thus the measurements, were performed with a Noise Level Analyzer, type Solo Master (01dB-Stell-ΜVI Technologies Group, France) and the outdoor microphone was used is MCE 212 (class 1).

The noise level analyzer accuracy conforms to IEC 61672-1 international standard, which represents sound level meters suitable for general field applications. The monitoring station used in combination with pre-amplifier, filter set and weighting network.

Also, a computer was used for initial setting up and checking the process of the recording metrics. The computer is equipped with the software responsible to collect and analyse the measurement data and presents the measurement results. Also, the instruments were equipped with noise statistical analyzers.

In order to monitor the credibility of the measurements through the duration of the recordings of the acoustic environment, the instruments were calibrated with an acoustical calibrator.

In accordance with the European and Greek legislation (Directive 2002/49/EC) the noise level analyzer was mounted on a tripod at a height of 4.0 ± 0.2 m above the ground and at a 2 m minimum resolution on each building facade.

Necessarily, a distance was taken between the sound level meter and the surrounding obstacles. The reason is that these obstacles, which include the building walls or building facades, may intensify or reduce the received noise level (Kelessis et al., 2005).

Furthermore, for the weather protection, the microphone is covered by a windscreen. Thus, the monitoring station can be left unattended for a long period without being damaged by the weather conditions such as rain, snow or wind.

**Fig. 3:**
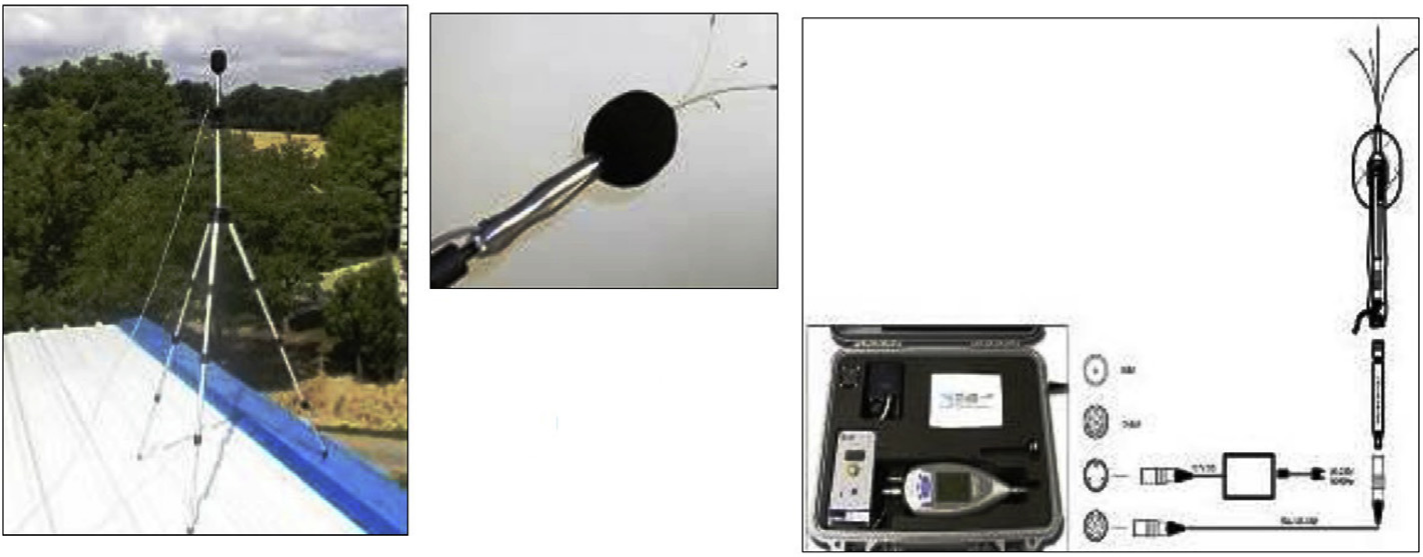
Noise level analyzer (Solo Master) and microphone (MCE 212).

In Fig. 3 the noise level analyzer and the microphone used for the measurement procedure are presented.

The wide noise measurement range allows the instrument to be used for a diverse range of environmental noise investigations.

In the environmental analysis, the sound levels fluctuate with time and thus the equivalent noise level (L_Aeq,T_) is must be measured.

The sound level meter records continuous steady noise levels during a 24-h period and automatically calculates the equivalent noise levels (L_Aeq,T_), according Equation (1).

(1) $$L_{Aeq,T}=10log_{10}\int_{0}^{T}\frac{P_{A\left(t\right)}^{2}}{P_{0}^{2}}dt$$

The L_Aeq,T_ represents the sound pressure level in decibels averaged over a certain period of the time (T) in terms of energy, with frequency ranges, being measured according to the sensitivity scale A of the human ear. The biophysical quantity A-weighting sound level, which expressed in A-weighted decibels (dB(A)), is the type of scale introduced to account the subjective nature of noise exposure. Therefore, the sound pressure levels rates at different frequencies in a way comparable to that of the human hearing (Passchier-Vermeer and Passchier, 2000).

From the L_Aeq,T_ measurements, we calculated the L_min_ and L_max_ values. The L_min_ and L_max_ represents the maximum and minimum values measured over the one year period of the road traffic noise measurements.

Moreover, according to the Directive 2002/49/EC the member states apply the noise indicators L_den_ and L_night_ for the assessment of harmful effects on human health and wellbeing.

Hence, we calculated the L_day_, L_evening_, L_den_ and L_night_ environmental noise indices.

In accordance with the Greek legislation the national limit values for the transportation noise are 70 dB and 60 dB, respectively, for the L_den_ and L_night_ environmental noise indicators.

From the measurements of equivalent noise levels we estimate the A-weighted day-evening-night equivalent sound level L_den_ in decibels (dB), calculated for the 24-h period of the day with the following equation:

(2) $$L_{den}=10\times \log\frac{1}{24}\left(12\times 10^{\frac{Lday}{10}}+4\times 10^{\frac{Levening+5}{10}}+8\times 10^{\frac{Lnight+10}{10}}\right)$$

in which:

L_day_ indicator is the A-weighted long-term average sound level, determined for 12 h of the day between 07.00 to 19.00 local time.

L_evening_ indicator is the A-weighted long-term average sound level, determined for the evening during 4 h between 19.00 to 23.00 local time.

L_night_ indicator is the A-weighted long-term average sound level, determined for 8 h during the night between 23.00 to 07.00 local time.

The 10 dBs penalty in the Equation (2) was added to the sound levels between 22.00 and 07.00, while the 5 dBs penalty was added to the levels between 19.00 and 22.00 to reflect individual's sensitivity to noise pollution during the night and the evening.

Therefore, we calculated the noise indicators L_den_ and L_night_ from the available noise measurements using Equation (2). The noise values for the indicators L_day_, L_evening_, and L_night_, as calculated in the Equation (2), collected from the 24-h measurements adjust for the mean values for the time period 07.00 to 19.00, 19.00 to 23.00 and 23.00 to 07.00, respectively.

